# Iris implantation cysts growth five decades after trauma

**DOI:** 10.1016/j.ajoc.2025.102333

**Published:** 2025-04-11

**Authors:** Colya N. Englisch, Karl T. Boden, Clemens N. Rudolph, Charlotte Semoulin, Peter Szurman, Philip Wakili

**Affiliations:** a Eye Clinic Sulzbach, Knappschaft Hospitals, 66280 Sulzbach/Saar, Germany; b Institute of Experimental Ophthalmology, Saarland University, 66421, Homburg, Germany; c Klaus Heimann Eye Research Institute (KHERI), 66280 Sulzbach/Saar, Germany

**Keywords:** Iris, Iris tumor, Iris cyst, Iris implantation cyst, Epithelial downgrowth, Trauma, Surgery

## Abstract

**Purpose:**

Implantation cysts of the iris are rare benign lesions resulting from epithelial downgrowth after trauma or surgery. This report highlights the management of an exceptional case of implantation cysts manifestation five decades after penetrating childhood trauma.

**Observation:**

A 59-year-old male patient with a history of penetrating trauma of the cornea at the age of 4 years presented with cystic anterior chamber lesions exhibiting pigment deposition. The pupil was distorted and almost entirely covered. 50 MHz ultrasound biomicroscopy and anterior segment optical coherence tomography displayed no evidence of malignancy. The cystic lesions were excised in an endothelium-protective manner and histopathological analysis revealed dysplasia-free corneal squamous epithelium compatible with implantation cysts of the iris.

**Conclusion and importance:**

Implantation cysts of the iris are benign and rare. Anterior segment imaging is required to exclude malignancies. Most cases emerge after surgery or trauma within months to a few years after injury. However, occurrence can also be delayed by several decades. It is thus mandatory to demand a full and long–lasting ophthalmological anamnesis, to accurately distinguish, diagnose, and treat cystic lesions of the iris.

## 1 Introduction

Iris tumors include solid entities and rarer cystic lesions.^1^ The current WHO classification distinguishes between pigmented epithelial cysts and implantation cysts.^2^ The former are usually of neuroepithelial origin, primary, and stationary, whereas implantation cysts are often secondary and progressive.^3^ Epithelial implantation may be cystic or diffuse in growth.^3^ Causes of epithelial implantation include surgery and trauma.^3^ These cysts tend to affect neighboring structures regularly leading to severe functional deficits.^3^

This is an exceptional rare case of iris implantation cysts manifestation five decades after penetrating childhood trauma of the cornea.

## 2 Case report

A 59-year-old male patient presented with increasing symptoms of haze for 2–3 years, which disappeared on reduction of light intensity. Ophthalmological history comprised an unspecified penetrating corneal trauma after playing in the sandbox at the age of 4 years. No specific details regarding the surgical management of the childhood trauma were known. Residual history of surgery or systemic treatment including chemotherapy, corticosteroids or ocular irradiation was absent. Past medical history was uneventful.

**Fig. 1:**
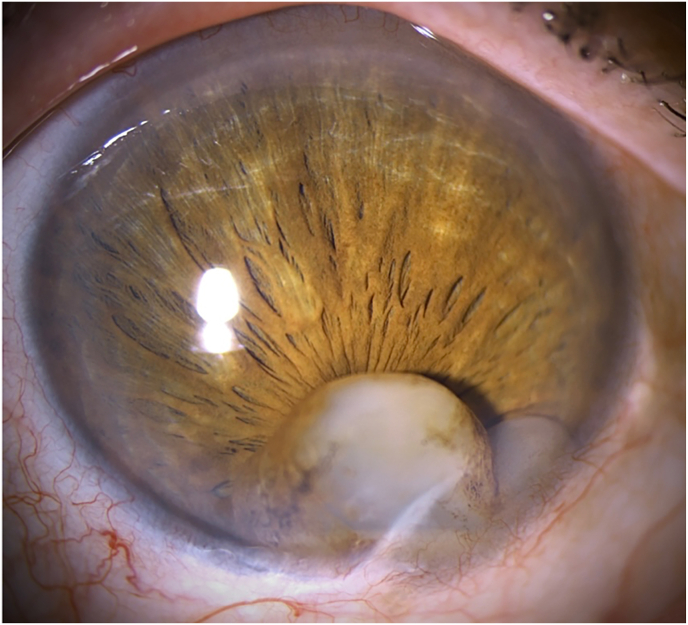
Slit lamp biomicroscopy of the corneal scar and implantation cysts of the iris preoperatively.

Slit lamp examination of the right eye revealed a 2 mm long whitish corneal scar inferiorly with an associated pair of anterior chamber cystic lesions with basal pigment deposition ranging from the iris to the corneal endothelium (Fig. 1). Respective dimensions of the cystic lesions, measured using a slit lamp, were 3.7 × 4.6 and 1.4 × 1.4 mm. The pupil was distorted towards 6 o'clock and almost completely covered by the cystic lesions. The fellow eye was normal. Visual acuity, assessed via autorefractometry in low-light conditions, was 0.2 (20/100) in the right eye and 1.0 (20/20) in the left eye.

**Fig. 2:**
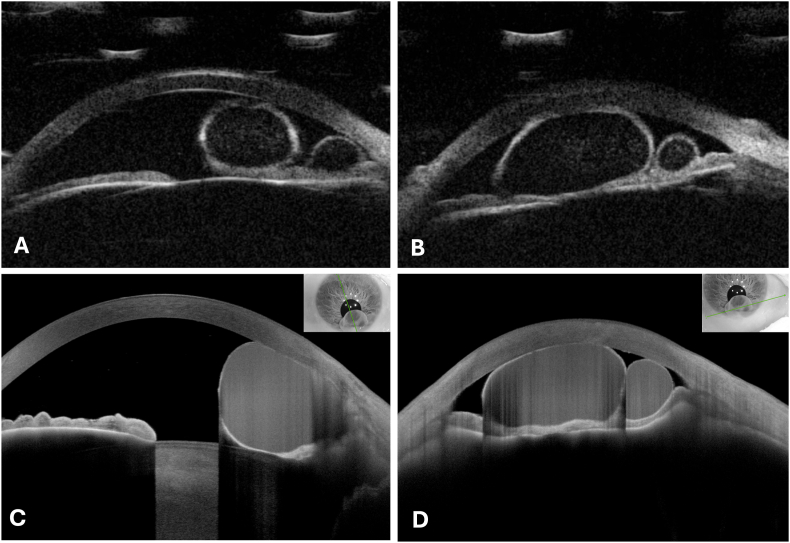
Ultrasound biomicroscopy (A and B) and optical coherence tomography (C and D) of the implantation cysts of the iris in a central (A and C) and peripherical (B and D) cross section of the cornea.

Ultrasound biomicroscopy (UBM, 50 MHz) visualized 2 roundshaped cystic lesions with thick hyperechogenic walls and hypoechogenic content (Fig. 2A and B). Anterior segment optical coherence tomography (OCT) displayed cystic lesions with thin hyperreflective walls and isoreflective content (Fig. 2C and D). Both had contact with the corneal endothelium. There was no evidence of malignancy such as solid masses, irregular borders, rough surfaces, or prominent vascularization supporting the suspected diagnosis of liquid-filled implantation cysts of the iris (Fig. 2).^4^

Surgery indication was given, also owing to the severe visual impairment. The cystic lesions were excised in an endothelium-protective manner. Briefly, viscodissection using cohesive viscoelastic was performed between the cyst and the endothelium using a “no-touch” technique. Subsequently, the cyst was sharply punctured, and its contents were aspirated using a 28G needle. The cyst wall was removed using crocodile forceps. Intraoperatively, a posterior subcapsular cataract was observed.

**Fig. 3:**
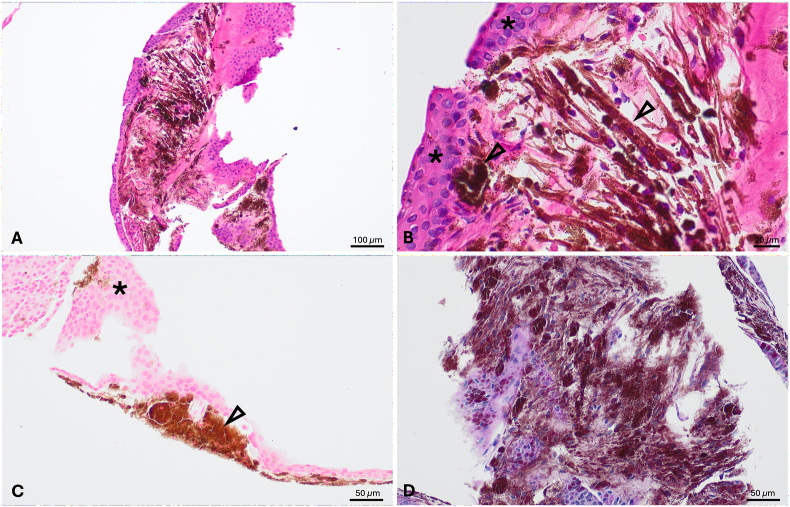
Hematoxylin & eosin staining: overview of the histological specimen, showing the wall of a cystic structure lined with uniform epithelium (A) and high magnification, featuring a stratified squamous epithelium with uniform nuclei and a brownish-black, finely granular pigment beneath it. (B). Periodic acid Shiff staining at high magnification (C). Iron Berlin blue staining showing a brownish-black pigment, indicating a negative result, as a positive result would appear blue. (D). The asterisk highlights layers of dysplasia-free squamous epithelium from the cyst wall, while the arrowheads point to iris pigment deposits. (For interpretation of the references to colour in this figure legend, the reader is referred to the Web version of this article.)

Histopathological analysis revealed a stratified squamous epithelium without dysplasia, consistent with implantation cysts originating from corneal epithelium (Fig. 3A and B). Periodic acid Shiff (PAS) staining gave no indication of goblet cells (Fig. 3C). Adjacent stroma exhibited fibrosis with pigment deposition originating from iris melanin, and not from iron derivatives as confirmed by negative iron Berlin blue staining (Fig. 3D).

**Fig. 4:**
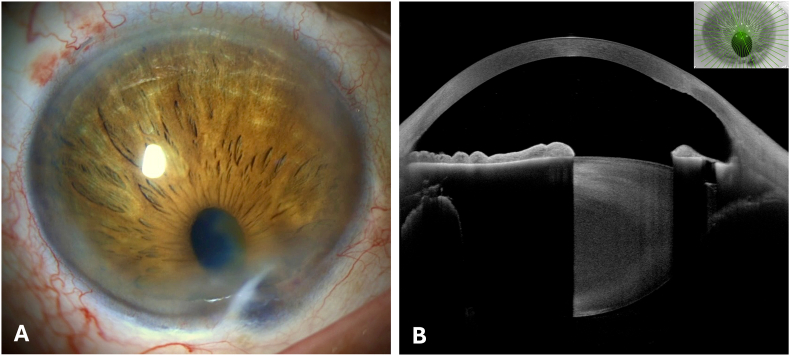
Postoperative findings. Slit lamp biomicroscopy (A) and anterior segment optical coherence tomography (B).

Fig. 4A and B shows the postoperative findings in slit lamp biomicroscopy and anterior segment OCT several weeks after surgical excision (Fig. 4).

## 3 Discussion

Epithelial implantation is rare.^3^ Implantation cysts of the iris result from downgrowth of ectopic epithelial cells of various histological origins (e.g. cornea, conjunctiva, etc.).^3^ The combination of ocular trauma or surgical history with clinical findings is essential for the diagnosis of secondary cysts.^3^ Of note, the concordant localization of the corneal scar and the cystic lesions in a restricted sector at 6 o'clock corroborates the diagnosis of epithelial implantation cysts of the iris.

Epithelial cell downgrowth into the anterior chamber during or shortly after trauma is considered to be causative.^3^^,^^5^^,^^6^ Under physiological conditions, foreign epithelial cells do not survive in the aqueous humor. However, alteration of the aqueous humor, such as occurs in severe disease or trauma, may allow for survival and promote implantation.^5^^,^^6^ Mechanisms of early intracorneal encapsulation and delayed epithelial cell release or generalized delayed accelerated cyst growth have been postulated to explicate iris cyst growth after several decades.^7^

Primary and secondary iris cysts can be distinguished. Primary iris cysts originate from the pigment epithelium or the stroma.^8^ Pigment epithelium cysts are a common and important differential diagnosis of iris neoplasms including iris and ciliary body melanoma,^9^, ^10^, ^11^ medulloepithelioma,^12^^,^^13^ and primary or metastatic carcinoma or lymphoma.^3^^,^^14^, ^15^, ^16^, ^17^, ^18^, ^19^, ^20^ They grow anteriorly to the iris pigment epithelium within the iris stroma commonly distorting the iris shape.^3^^,^^21^

Next to the common implantation cysts, secondary iris cysts also account for uveitic (e.g., nongranulomatous uveitis), parasitic, tumor-induced (i.e., metastases or primary iris tumors), drug-induced (e.g., myotics, latanoprost), and systemic disorder-associated cysts (e.g., diabetes mellitus, mucopolysaccharidoses, Menkes syndrome, malignancies).^3^

In contrast to primary iris cysts, secondary forms display a more aggressive expansion behavior, thus more frequently leading to complications.^3^ In UBM, implantation cysts will often appear with a thick wall, stromal cysts with a thin.^3^ In histology, implantation cysts are difficult to distinguish from stromal cysts. The epithelial lining of implantation cysts consists of concentric layers of stratified squamous epithelium, whereas variations from stratified squamous to unilayered cuboidal are described in stromal cysts.^3^^,^^22^ Implantation cysts can be pigmented as in the here presented case, whereas stromal cysts are usually not pigmented.^23^ Also, goblets cells, which were not detected herein, are commonly found in iris stromal cysts but not in implantation cysts originating from the cornea.^22^^,^^24^ Thus, the clinical association between the history of trauma, the presence of a corneal scar anteriorly to cyst formation, and the histological features of the cyst wall that are distinct from iris stromal cysts but consistent with implantation cysts provides compelling evidence supporting our diagnosis of implantation cyst.

Iris cysts require evaluation with anterior segment imaging including high-resolution UBM and OCT to exclude differential diagnoses.^3^ Metastases, endothelial proliferation, and iridocorneal endothelial syndrome are important differential diagnoses of implantation cysts.^3^

Iris cysts treatment is indicated depending on functional deficits. Therapeutic strategies follow a conservative trend ranging from observation towards laser treatment, fine-needle aspiration, and surgical excision as ultima ratio.^3^ Absolute alcohol-induced sclerosis has also been performed with good clinical results.^25^^,^^26^ Recurrence is regularly observed, for instance when the cyst wall is not entirely excised or when epithelial cell spreading has occurred.^5^

Behrouzi and Khodadoust reported implantation cyst growth within a period between 3 months and 9 years after injury in an impressive series of 102 cases collected within a timeframe of 18 years.^25^ Borella et al. recently presented a case of cyst manifestation 24 years after penetrating keratoplasty.^14^ This evidences the exceptional character of this case of late cyst growth more than five decades after trauma, which is to the authors’ best knowledge the latest in detail described manifestation after injury.

In conclusion, implantation cysts of the iris are benign and rare, emerge after surgery or trauma, and require anterior segment imaging to exclude malignancies. Cyst growth mostly occurs within months to few years after injury. However, as this unique case highlights, it can also be delayed by several decades. It is thus mandatory to demand a full and long–lasting ophthalmological anamnesis, to accurately distinguish, diagnose, and treat cystic lesions of the iris.

## CRediT authorship contribution statement

**Colya N. Englisch:** Writing – original draft, Visualization, Investigation, Data curation, Conceptualization. **Karl T. Boden:** Writing – review & editing, Resources, Conceptualization. **Clemens N. Rudolph:** Writing – review & editing, Data curation. **Charlotte Semoulin:** Writing – review & editing, Data curation. **Peter Szurman:** Writing – review & editing, Resources, Conceptualization. **Philip Wakili:** Writing – review & editing, Data curation, Conceptualization.

## Declarations and patient consent

The study adhered to the tenets of the Declaration of Helsinki. Anonymous case reports are waived by the local Institutional Review Board (Ethikkommission bei der Ärztekammer des Saarlandes). The patient formally assigned the written consent for publication.

## Authorship

All authors attest that they meet the current ICMJE criteria for Authorship.

## Funding

No funding or grant support.

## Declaration of competing interest

The authors declare that they have no known competing financial interests or personal relationships that could have appeared to influence the work reported in this paper.

## List of abbreviations

OCT: optical coherence tomography; PAS: Periodic acid Schiff; UBM: ultrasound biomicroscopy; WHO: world health organization.
